# Retrovirus reactivation in *CHMP2B*^*Intron5*^ models of frontotemporal dementia

**DOI:** 10.1093/hmg/ddaa142

**Published:** 2020-07-06

**Authors:** Laura Fort-Aznar, Chris Ugbode, Sean T Sweeney

**Affiliations:** 1 Department of Biology, University of York, York YO10 5DD, UK; 2 York Biomedical Research Institute, University of York, York YO10 5DD, UK

**Keywords:** retrovirus, frontotemporal dementia, amyotrophic lateral sclerosis, *Drosophila*, *gypsy*

## Abstract

Frontotemporal dementia (FTD) is the second most prevalent form of pre-senile dementia after Alzheimer’s disease. Amyotrophic lateral sclerosis (ALS) can overlap genetically, pathologically and clinically with FTD indicating the two conditions are ends of a spectrum and may share common pathological mechanisms. FTD–ALS causing mutations are known to be involved in endosomal trafficking and RNA regulation. Using an unbiased genome-wide genetic screen to identify mutations affecting an FTD–ALS-related phenotype in *Drosophila* caused by CHMP2B^Intron5^ expression, we have uncovered repressors of retrovirus (RV) activity as modifiers of *CHMP2B^Intron5^* toxicity. We report that neuronal expression of CHMP2B^Intron5^ causes an increase in the activity of the endogenous *Drosophila* RV, *gypsy*, in the nervous system. Genetically blocking *Drosophila gypsy* activation and pharmacologically inhibiting viral reverse transcriptase activity prevents degenerative phenotypes observed in fly and rat neurons. These findings directly link endosomal dysfunction to RV de-repression in an FTD–ALS model without TDP-43 pathology. These observations may contribute an understanding to previous discoveries of RV activation in ALS affected patients.

## Introduction

Frontotemporal dementia (FTD) is the second most common form of dementia in individuals under 60 years of age (1). FTD is characterized by atrophy of the frontal and temporal lobes. Within these brain regions, von Economo neurons are first affected triggering behavioral changes and cognitive impairment (2). FTD can co-occur with amyotrophic lateral sclerosis (ALS) and both conditions share clinical, neuropathological and genetic features, suggesting they may constitute two ends of a disease continuum (3–5) with approximately 15% of FTD patients developing ALS and vice versa (6).

Mutations in chromatin-modifying protein 2B (CHMP2B) have been found to cause rare cases of FTD (7,8), but have also been identified in some ALS cases (9–11) and in FTD–ALS (12). CHMP2B encodes a core component of the endosomal sorting complex required for transport-III (ESCRT-III) involved in transport of ubiquitinated proteins from the cell membrane to the lysosome (13). The pathological FTD-causing *CHMP2B^Intron5^* mutation arises from a C-terminal truncation of the protein, perturbing endosomal trafficking resulting in autophagosome accumulation leading to neurodegeneration. The *CHMP2B^Intron5^* mutation causes a form of FTD with no apparent TAR DNA-binding protein 43 (TDP-43), FUS or tau proteinopathy (14). Previous studies have established a *Drosophila* model of *CHMP2B^Intron5^* (15), which has facilitated the unbiased screening for genetic modifiers of *CHMP2B^Intron5^* toxicity identifying innate immune activation, autophagosomal and endosomal dysfunction (15–18). Here, we describe the identification of retrovirus (RV) reactivation as a potent modifier of the *CHMP2B^Intron5^* phenotype.

Endogenous RVs are present in most eukaryotic genomes, accumulating in heterochromatic regions (19). They can act as genetically mobile elements that can replicate and move within the genome negatively affecting host health. RVs contain three open reading frames (ORFs) consisting of *group-specific antigen* (*GAG*), *polymerase* (*POL*) and *envelope* (*ENV*) components that upon activation, encode the capsid, reverse transcriptase and ENV protein of the virus, respectively. RVs can be initially repressed via cellular antiviral mechanisms and later domesticated and endogenized within the genome over time, with gain-and-loss of ORFs and ORFs evolving to generate novel proteins (20). In order to prevent RV activation, transposition and infection, eukaryotic cells have developed potent RV suppression mechanisms: small RNA molecules associated with the piwi protein termed piRNAs (21,22), which are known to act in the germline, and siRNAs that have a somatic role (23). An increase in RV expression has been documented in FTD–ALS postmortem tissue (24–26) suggesting that the RV silencing machinery might be disrupted potentially through TDP-43 dysfunction (27–29). An increase in the reverse transcriptase activity of human endogenous RV (hERV)-K has been documented in ALS patient serum and cerebrospinal fluid but the link between ALS pathology and RV abundance has yet to be fully identified (30). Neuronal expression of the hERV-K ENV protein in mice induced an ALS-like condition with loss of upper- and lower-motor neurons (26). These findings have been followed by clinical studies using nucleoside reverse transcriptase inhibitors as potential therapeutic treatments for ALS (31). Why RVs might be de-repressed in ALS tissue in some patients and if they may contribute to pathology has yet to be fully elucidated.

We have identified mutations in genes functioning in RV repression as dominant genetic enhancers and suppressors of CHMP2B^Intron5^ expression in *Drosophila* eye. Mutations, knockdown or overexpression of the RV repression component genes encoding *Brother-of-Yb (BoYb), piwi, Sister-of-Yb (SoYb)* and *Vreteno* (*Vret*) are seen to enhance and suppress an innate immune activation eye phenotype associated with CHMP2B^Intron5^ expression (15). Quantification of RV insertions in genomic DNA from flies neuronally expressing CHMP2B^Intron5^ shows an elevation of *gypsy* elements inserted in the genome with a concomitant increase in expression of the *gypsy* ENV protein. We show that knockdown of ENV protein via *gypsy*-RNAi in the presence of *CHMP2B^Intron5^* alleviates photoreceptor cell death. Finally, we demonstrate that the addition of reverse transcriptase inhibitors in primary mammalian neurons transfected with *CHMP2B^Intron5^* significantly rescues the neuronal dendritic retraction caused by CHMP2B^Intron5^ expression.

## Results

### A genetic screen in *Drosophila* identifies RV repression components as modifiers of *CHMP2B*^*Intron5*^ toxicity

**Figure 1:**
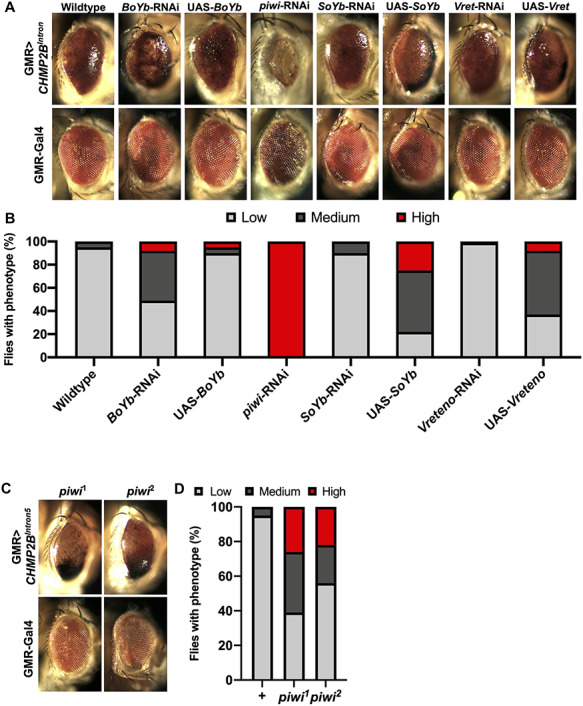
Manipulating levels of *BoYb*, *piwi*, *SoYb* and *Vret* perturbs melanization in the *CHMP2B^Intron5^* expressing *Drosophila* eye. Representative eye of *GMR*-Gal4:UAS-*CHMP2B^Intron5^*/+ (*GMR* > *CHMP2B^Intron5^*) (**A**). *BoYb* knockdown via RNAi showed an enhancement on the eye blackening, whereas overexpression rescued the melanization caused by *CHMP2B^Intron5^* expression. Reduction of *piwi* levels via *Piwi*-RNAi strongly increased the *CHMP2B^Intron5^* eye melanization. A single copy of *SoYb*-RNAi and *Vret*-RNAi reduced the toxicity on the eye caused by *CHMP2B^Intron5^*, whereas *SoYb* and *Vret* overexpression enhanced the *CHMP2B^Intron5^* melanization phenotype. (**B**) Quantification of the eye melanization severity from A genotypes (*n* = 100). (**C**) A single copy of the mutant alleles *piwi^1–2^* enhanced the toxicity of the eye melanization caused by CHMP2B^Intron5^ expression. (**D**) Quantification of the eye melanization severity from C genotypes (*n* = 100).

The *CHMP2B^Intron5^* mutation was identified in a large Danish family causing FTD on chromosome 3 [FTD-3; (7)]. In this study, we overexpressed this human mutant form in the *Drosophila* eye using the *glass multimer reporter* (*GMR*) promoter. This induces a rough eye with melanotic deposits [Fig. 1A; (15)]. The melanotic spots represent an innate immune activation and we principally quantify the level of melanization in our screen as a measure of FTD–ALS-related neuronal death and dysfunction (15). This model has identified several modifiers of *CHMP2B^Intron5^* (15–18). From a genome-wide unbiased screen for enhancers and suppressors of this phenotype, we identified a heterozygous mutation in *BoYb* as a dominant genetic enhancer of *CHMP2B^Intron5^* related toxicity (Fig. 1A and B). *BoYb* knockdown via RNAi potentiated the *CHMP2B^Intron5^* melanization phenotype whereas overexpression reduced the melanization of the eye caused by *CHMP2B^Intron5^* (Fig. 1A). BoYb is a component of the Yb-nuage piRNA regulatory machinery (32). To confirm and extend this data *in vivo*, a targeted screen using mutations and transgenes for other components of the RV repression machinery was performed to test for dominant modification of the *CHMP2B^Intron5^* eye expression phenotype. Novel enhancers and suppressors identified included *piwi, SoYb* and *Vret* [Fig. 1A–D; (33)]. Enhancement of melanization by *piwi* was consistent for two additional identified *piwi* mutant alleles (Fig. 1C and D). Each of these are core components of the *Drosophila* Yb-nuage complex involved in RV repression (34). In control flies driving *GMR*-Gal4, these crosses had minimal effects on eye degeneration (Fig. 1A).

### Neuronal CHMP2B^Intron5^ expression leads to *gypsy* activation

Having identified a genetic interaction between piRNA regulatory machinery and CHMP2B^Intron5^ expression in the fly eye, the molecular pathways involved in RV upregulation in the *Drosophila* FTD–ALS model expressing CHMP2B^Intron5^ were investigated. Levels of several RV genomic elements were examined by quantitative polymerase chain reaction (qPCR) in heads of flies expressing *GMR* > *CHMP2B^Intron5^*. Only insertions of the endogenous *Drosophila* RVs *gypsy, gypsy-6* and *tirant* were observed to be significantly upregulated in the genome of *GMR* *> CHMP2B^Intron5^* fly heads, with *gypsy* being the most strongly increased in abundance compared with wild-type and *ago2* mutants, *AGO2^454/321^* (Supplementary Material, Fig. S1A), a positive control known to regulate RVs (35,36). Individual RV elements can degenerate and lose ORFs from the RV element, but still be mobilized in a non-autonomous manner by intact RVs. To examine the abundance of degenerate *gypsy* elements in the presence of *CHMP2B^Intron5^*, we quantified the *gypsy*-genome components *GAG*, *POL, ENV* and *LTR* copy levels in the *CHMP2B^Intron5^* expressing genome (Supplementary Material, Fig. S1B). A significant increase was observed only for the *ENV* (Fig. 1A and Supplementary Material, Fig. S1B) and *LTR* (Supplementary Material, Fig. S1B) sequences in the *CHMP2B^Intron5^* expression background compared with the wild-type indicating a mobilization of a degenerate *gypsy* element containing *ENV*.

**Figure 2:**
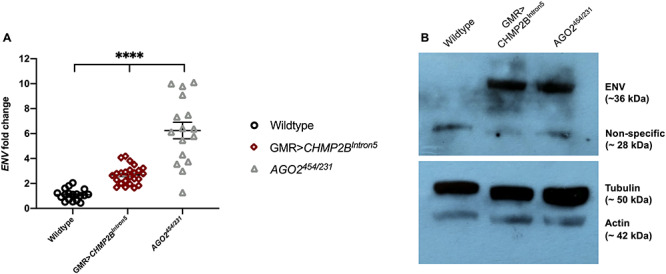
Increased *gypsy*-ENV expression is detected in pan-neuronally expressing *CHMP2B^Intron5^* Drosophila. (**A**) Fold change in expression of *gypsy-ENV* measured in wild-type (+), *GMR-Gal4:UAS-CHMP2B^Intron5^* (*GMR* > *CHMP2B^Intron5^*) and *AGO2^454/231^* mutant fly heads by qPCR. Transcript levels are normalized to Rpl32 and fold-change values are relative to wild-type flies. Error bars indicate standard error (Kruskal–Wallis test, ^****^P < 0.0001). (**B**) Immunoblot showing ENV levels in wild-type, *GMR* > *CHMP2B^Intron5^* and *AGO2^454/321^* adult heads.

Given the increase of *gypsy-ENV* sequence in *CHMP2B^Intron5^* flies (Fig. 2A), ENV protein levels in CHMP2B^Intron5^ expressing tissue were then examined. Immunoblotting using an anti-*gypsy*-ENV antibody (37) confirmed ENV to be present in *CHMP2B^Intron5^* expressing and *AGO2^454/321^* mutant heads in comparison to the wild-type heads (Fig. 2B), indicating a significant expression of the *gypsy*-ENV protein in the presence of *CHMP2B^Intron5^*.

### *Gypsy* mobilization is observed *in vivo* in CHMP2B^Intron5^ expressing neurons

**Figure 3:**
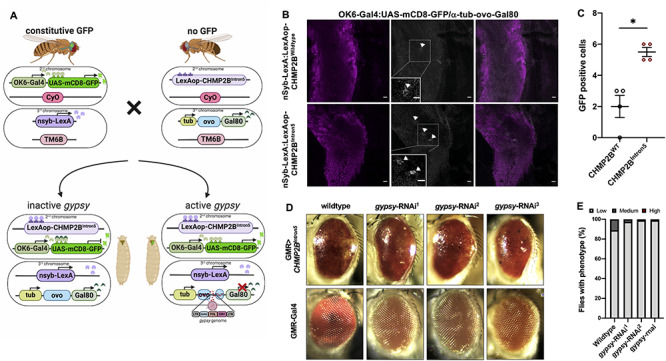
The *gypsy*-TRAP reporter confirms increased *de novo* insertion of *gypsy* in CHMP2B^Intron5^ expressing *Drosophila* brains. (**A**) Schematic of the *gypsy*-TRAP mobilization assay. De-repressed *gypsy* jumps into the hotspot *ovo* binding site and disrupts Gal80, triggering GFP expression in motor neurons. Image created with BioRender. (**B**) Pan neuronally driven (nSyb-LexA) *CHMP2B^Intron5^* brains show increased GFP-positive cells compared with pan neuronally driven *CHMP2B^Wildtype^* brains. GFP-positive cells represent *gypsy* mobilization events, which are limited to the motor neurons because of the OK6-Gal4 driver. Scale bar, 10 μm. (**C**) Quantification of GFP-labeled neurons observed in nSyb > *CHMP2B^Wildtype^* and nSyb > *CHMP2B^Intron5^* expressing *Drosophila* brains (*t* test, ^*^P < 0.01). (**D**) Inhibition of *gypsy* via three different *gypsy*-RNAi (1, 2 and 3) rescued the degenerative eye phenotype caused by *GMR-Gal4:UAS-CHMP2B^Intron5^* (*GMR* > *CHMP2B^Intron5^*). (**E**) Quantification of the eye phenotypes from (D) genotypes (*n* = 100).

To test whether *gypsy* is an active RV transposing physically in the presence of *CHMP2B^Intron5^*, we employed a reporter system based on RV integration into a genomic RV landing hotspot, known as *ovo* (38). We used a reporter system termed *gypsy*-TRAP to detect *de novo* insertions of *gypsy* [Fig. 3A; (28)]. In this system, a promoter-Gal4 construct is used to drive GFP in a tissue of interest, here OK6-Gal4, which expresses in motor neurons. In the same fly, Gal80 is expressed under the control of an α-*tubulin*-promoter repressing Gal4 in all tissues. Between the α-*tubulin*-promoter and the Gal80 element there is a section of the *ovo* promoter, a genomic hotspot for *gypsy* insertion events (37). If *gypsy* elements jump into the intervening hotspot, Gal80 expression is reduced and Gal4 is de-repressed, driving a GFP signal in motor neurons (Fig. 3A and B). To allow both *CHMP2B^Intron5^* and *CHMP2B^Wildtype^* expression in combination with the *gypsy*-TRAP system, a Gal4 independent expression system was employed. To achieve this, the LexA/LexAop system (39) was used to express CHMP2B^Wildtype^ and CHMP2B^Intron5^. To ensure genetic consistency for the initial number of genomic *gypsy* insertions prior to CHMP2B induced activation, both constructs were genomically site landed to ensure identical genomic backgrounds and equivalent expression levels of both CHMP2B^Wildtype^ and CHMP2B^Intron5^ proteins. Transposition was assayed in motor neurons of pan-neuronal expression of CHMP2B^Wildtype^ or CHMP2B^Intron5^ via nSyb*-*LexA (Fig. 3B). In the presence of Gal80 and absence of CHMP2B^Intron5^ expression, GFP is silenced and few motor neurons are labeled. When CHMP2B^Intron5^ is neuronally expressed, we observed GFP expression in multiple single motor neurons (Fig. 3B and C). In the presence of *CHMP2B^Intron5^*, the *gypsy*-TRAP highlights a significant increase in *gypsy* mobilization in CHMP2B^Intron5^ expressing cells compared with CHMP2B^Wildtype^ (Fig. 3B and C). Together with the findings described above, these results demonstrate that *gypsy* activity is higher in CHMP2B^Intron5^ expressing *Drosophila* neurons.

Having identified an upregulation of *gypsy*-ENV and *gypsy*-mediated transposition events in CHMP2B^Intron5^ expressing neurons, we then asked whether it was possible to rescue the melanized eye phenotype caused by *GMR* > *CHMP2B^Intron5^* via *gypsy* downregulation (Fig. 3D and E). Three different RNAi constructs targeting *gypsy* were tested for their ability to reverse melanization in fly eyes expressing *CHMP2B^Intron5^* (40). Reducing *gypsy* via the three different RNAi constructs alleviated the melanization phenotype in the fly eye caused by CHMP2B^Intron5^ expression (Fig. 3D and E).

### Inhibition of reverse transcriptase rescues neuronal aberrations caused by mammalian CHMP2B^Intron5^ expression

**Figure 4:**
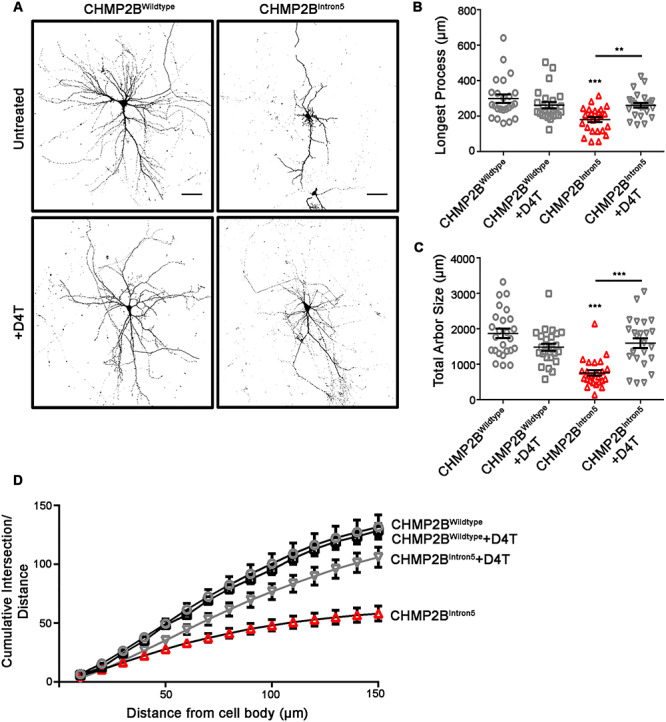
The retroviral reverse transcriptase inhibitor Stavudine (D4T) rescues dendritic collapse in mammalian neurons expressing CHMP2B^Intron5^. (**A**) Representative micrographs of mature neurons expressing FLAG-tagged *CHMP2B^Wildtype^* or *CHMP2B^Intron5^* in the absence and presence of D4T (Stavudine). Scale bar, 50 μm. Longest process length (**B**) and total arbor size (**C**) in CHMP2B^Intron5^ expressing neurons are significantly increased upon addition of D4T. (**D**) *CHMP2B^Intron5^* presents a reduced cumulative branch number partially rescued by D4T. (B–D) One-way ANOVA with Tukey’s post-hoc comparing *CHMP2B^Intron5^* and without D4T (^**^P < 0.01, ^***^P < 0.001).

Having observed an amelioration of the melanized eye phenotype in *Drosophila* when *gypsy* was inhibited, we asked whether similar effects could be recapitulated in mammalian models. Neurons transfected with CHMP2B^Intron5^ develop a significant retraction of the dendritic arbor when compared with CHMP2B^Wildtype^ expressing controls [Fig. 4; (18)]. Stavudine (D4T) and lamivudine (3TC) are nucleoside analogue reverse transcriptase inhibitors that are widely used to inhibit human immunodeficiency virus replication in patients and are well tolerated as an anti-retroviral therapy (41). Primary neurons transfected with CHMP2B^Intron5^ showed a significant dendritic collapse phenotype compared with neurons transfected with CHMP2B^Wildtype^. CHMP2B^Intron5^ expressing neurons develop a significant reduction in the length of the longest neuronal process (Fig. 4B, 40% reduction) and a reduction in total arbor size (Fig. 4C, 60% reduction) when compared with CHMP2B^WIldtype^ expressing controls. This reduced complexity of the dendritic arbor was further analyzed using Sholl analysis, with a significant decrease in the cumulative number of intersections observed compared with wild-type controls (Fig. 4D). Administration of 10 μm of D4T (Stavudine) (48 h; Fig. 4) was sufficient to rescue these altered dendritic phenotypes. This observation was further confirmed with the administration of 10 μm 3TC (Lamivudine) (Supplementary Material, Fig. S2).

We observe that manipulation of cellular RV silencing machinery rescues innate immune related melanization phenotypes in the *Drosophila* eye caused by CHMP2B^Intron5^ expression. Additionally inhibition of retroviral reverse transcriptase in mammalian neurons expressing *CHMP2B^Intron5^* rescues dendritic collapse. Inhibition of *gypsy* via RNAi and administration of retroviral reverse transcriptase inhibitors D4T or 3TC was able to alleviate neurodegeneration-related phenotypes compared with controls (Figs 3D and E, Fig. 4; Supplementary Material, Fig. S2). Together, these data suggest that RV activity is increased in our *Drosophila* model and confirm that suppression of RV activation in both *Drosophila* and mammalian neurons reduces neurotoxicity, indicating RV suppression would be an attractive target for FTD–ALS treatment.

## Discussion

Our observations using *Drosophila* as a model for the FTD-causing mutation *CHMP2B^Intron5^* support an activation of the endogenous RV *gypsy* in CHMP2B^Intron5^ expressing *Drosophila* brains. Here, the RNA-silencing machinery Yb components, including BoYb, SoYb, Vret and piwi, were identified as dominant modifiers of CHMP2B^Intron5^ toxicity. Recent reports have found levels of RV encoded reverse transcriptase activity in serum and cerebrospinal fluid of FTD–ALS patients (30,42–45). Other studies have found in postmortem ALS tissue the presence of ENV, encoded by hERV-K, a human RV akin to *Drosophila-gypsy* (26,46). These findings have been replicated in a TDP-43 expressing *Drosophila* model of FTD–ALS (28,47). In this investigation, *gypsy*, *gypsy-6* and *tirant* insertions were increased in the presence of CHMP2B^Intron5^ among other RVs suggesting differential regulation of RVs. We focused our study on the well characterized RV *gypsy*. Here, a significant increase in *gypsy*-*ENV* transcript, as well as an upregulation of the glycoprotein ENV, is found in CHMP2B^Intron5^ expressing *Drosophila* heads. An increase in RV transcripts identified through a transcriptome analysis from *C9orf72*-affected patients has been previously observed (48). In an *in vitro* study to confirm hERV-K-ENV pathogenicity, the hERV-K-*ENV* ORF was transfected into human neuronal cells, which induced a decrease in cell number (26). In the same study, a transgenic mouse expressing hERV-K-ENV protein under a neuronal promoter was constructed and these animals developed an evident motor neuron degeneration, DNA damage and cortical thinning of the rostral part of the motor cortex (26). This suggests that the activation of these elements may be conserved and present among different FTD–ALS-causative mutations, though not all patients show elevated levels of hERV-K. RV elements have been characterized as TDP-43 targets at the DNA level (47) and an increase in RV expression found in ALS patients correlates with TDP-43 pathology (49). Although *CHMP2B^Intron5^* patients do not have apparent TDP-43 inclusions in the cytoplasm (12), manipulation of functionally associated ESCRT proteins can cause TDP-43 aggregation (50). We therefore cannot rule out a role for compromised TDP-43 function in CHMP2B^Intron5^ associated RV re-activation.

The suppression of RVs through reverse transcriptase inhibitors can ameliorate neurological defects associated with aging and FTD–ALS in *Drosophila* models (47,51). Here, we provide evidence that *gypsy* knockdown alleviates neuronal defects in our *Drosophila* model of FTD caused by the *CHMP2B^Intron5^* mutation. In our rat primary neuron model of FTD caused by CHMP2B^Intron5^ expression, reverse transcriptase inhibition is proposed to block mRNA production from RV elements via the inhibition of POL activity and the subsequent production of further GAG, POL and ENV proteins. Using two clinically well-characterized antiviral reverse transcriptase inhibitors, stavudine (D4T) and lamivudine (3TC), we were able to prevent the development of dendritic collapse in rat primary neurons expressing CHMP2B^Intron5^ (Fig. 4; Supplementary Material, Fig. S2). Preventing RV mobilization may help to limit neuronal and/or glial cell death in FTD–ALS. Recent studies have pointed to a role for TDP-43 in repressing RVs and retrotransposons (RTs), which also act via reverse transcriptase POL activity, both in human and *Drosophila* DNA (47,51–53). Around 50% of FTD patients do not have TDP-43 misregulation while 97% of ALS patients have TDP-43 aggregation (54,55), suggesting that only TDP-43-positive patients would benefit from RV inhibition. *CHMP2B^Intron5^*-affected patients are TDP-43-negative (7). In our *Drosophila* model expressing CHMP2B^Intron5^, RV disruption may be caused by other factors and not only by TDP-43 disturbance. Several RNA binding proteins (RBPs) linked to ALS, including MATR3, TDP-43, TIA1, hnRNPA1 and TAF15, are found to bind and regulate the RT *LINE1* RNA elements (56). This suggests that disruption of RBP function might dysregulate RV/RT repression. Thus, *CHMP2B^Intron5^*-affected patients could also benefit from RV inhibition, as our data suggests. Our work provides functional evidence for a conserved mechanism between *Drosophila* and mammalian models of FTD–ALS where RV inhibition provides an amelioration of the neurodegenerative condition via mechanisms that are yet to be discovered.

## Materials and Methods

### Fly stocks and maintenance

Flies were raised at 25°C on standard yeast, sugar and agar medium on a 12 h light:dark cycle. Unless otherwise stated, Canton-S was used for control. The following *Drosophila* lines were obtained from the Bloomington Stock Center, Indiana: UAS-*BoYb* (BL#28446), UAS-*BoYb*-RNAi (BL#62432), *piwi^1^* (BL#43637), *piwi^2^* (BL#43319), UAS*-piwi-*RNAi (BL#33724), UAS-*SoYb* (BL#26961), UAS-*SoYb*-RNAi (BL#36881), UAS-*Vret* (BL#22204), UAS-*Vret*-RNAi (BL#38212), *AGO2^454^* (BL#36522), *AGO2^321^* (BL#36511), *glass multiple reporter GMR*-Gal4 (BL#1104), *neuronal synaptobrevin* (nSyb)-LexA (BL#52817), OK6-Gal4 (Cahir O’Kane, University of Cambridge, UK), UAS-mCD8-GFP (BL#32186). For the genetic screen, lines containing *GMR-Gal4* and *UAS-CHMP2B^Intron5^* were recombined into the second chromosome as described elsewhere (15). *gypsy*-TRAP transgenic flies were a gift from Josh Dubnau (28). *gypsy*-RNAi transgenic flies were obtained from Jeng Pin (40). For flies used in gDNA extraction experiments, *UAS-CHMP2B^Intron5^* and *UAS-CHMP2B^Wildtype^* (15) were maintained in the same *w^1118^* background and crossed to *GMR*-GAL4 to ensure identical genetic backgrounds.

### Genetic interaction studies

To quantify the *CHMP2B^Intron5^* eye phenotype, the classification was divided into low, medium or high levels of melanization. *GMR*-GAL4::UAS-*CHMP2B^Intron5^* (*GMR* > *CHMP2B^Intron5^*) virgin females were crossed to mutant and transgenic stocks. Eyes were imaged using a camera (AxioCam Erc 5 s; Carl Zeiss, Germany) on a dissecting scope (Stemi 2000-C; Carl Zeiss).

### Generation of transgenic fly lines

To generate LexAop-*CHMP2B^Wildtype^* and LexAop-*CHMP2B^Intron5^* transgenic flies, the primers listed in Supplementary Material, Table S1 were used to amplify *CHMP2B^Intron5^* and *CHMP2B^Wildtype^* and cloned into a pJFRC19-13XLexAop2-IVS-myr::GFP vector (57) (Addgene, USA, Cat No. 26224) via the *BglII* and *Xba1* cloning sites. Constructs were then sequenced and inserted into the *Drosophila* genome at the *attP40* insertion site via *C31*-mediated site-specific attP integration. Integration at the attP40 site on the second chromosome was achieved by microinjection into the stock: *y,w,M(eGFP, vas-int, dmRFP)ZH-2A; P{CaryP}attP40*. Microinjections were carried out by the Cambridge Microinjection Service (UK).

### Immunohistochemistry

*Drosophila* immunohistochemistry was performed as described elsewhere (17). Primary antibodies used were: mouse anti-elav (DSHB 9F8A9, 1:50) and FluoTag®-X4 anti-GFP (NanoTag Biotechnologies, Germany, 1:1000). Primary antibodies were incubated with preparations overnight at 4°C. The following day, after washing with PBT they were incubated with the appropriate secondary antibodies for 1 h at room temperature before washing with PBT and mounting with Vectashield Mounting Medium (Vector Labs, USA). Control larvae were stained in the same solutions as experimental larvae. For rat primary neuron immunohistochemistry, cells were fixed and stained as previously described (58) using anti-FLAG (Sigma, USA, M2 clone, 1:1000). Primary antibodies were incubated overnight at 4°C. Corresponding Alexafluor secondary antibodies (Thermo Scientific, USA, 1:500) were incubated for 1 h at room temperature before mounting with Fluoromount (Sigma).

### Confocal microscopy and image analysis

For imaging *Drosophila* tissue, confocal images were acquired using a ZEISS LSM 880 confocal microscope using a 20× or 40× objectives using Zeiss filter sets for Alexa 488/561/633. Z-stack images of larvae brains were obtained using 20× or 40× NA oil objective. Images stacks were merged and processed using Fiji. For imaging rat primary neurons, images were collected on an inverted Zeiss microscope (880) with 20× Plan Neofluar objectives using Zeiss filter sets for DAPI and Alexa 488/546. Images were taken at an aspect ratio of 1024 × 1024. Images of neurons were traced using the NeuronJ plugin in ImageJ (1.6.0). Individual traces were saved, thresholded and Sholl analysis was conducted using the Sholl plugin.

### qPCR data analysis

gDNA was extracted using Puregene Core Kit A (Qiagen). SYBR® green assays were used to perform the qPCR analysis on a QuantStudio3 Real Time PCR System (Thermo Fisher Scientific, USA). Samples were run in triplicate and normalized to *rpl32* expression. Relative expression of genes was determined by the 2^−∆∆Ct^ method (59). Primers are listed in Supplementary Material, Table S1.

### Protein extraction and immunoblot

Fifty adult fly heads were homogenized in 30 μL of 2× Laemmli loading buffer. After boiling, samples were run on a 4–20% Mini-PROTEAN® TGX™ Precast Protein Gels (Biorad, USA) and transferred to PVDF membrane (Invitrogen, USA). Primary antibodies used include: mouse anti-ENV (37), anti-ß-Actin (Proteintech, USA, 60008–1-Ig, 7D2C10, 1:8000) and anti-ß-tubulin E7 (DSHB, 1:5000).

### Culture of primary neurons

Timed-mated female Wistar rats (Charles River, UK) (RRID:RGD_737929) were maintained in accordance with the UK Animals (Scientific Procedures) Act (1986). Cortices were dissected from postnatal day 1 (P1) mixed sex rat pups. Animals were euthanized using pentobarbital injection followed by cervical dislocation, according to Home Office guidelines. Cortical cell suspensions were obtained as previously (60) described and cytosine arabinoside (AraC, 2.4 μm final concentration) was added to the growth medium at 1 days *in vitro* (DIV). Neurons were transfected at 12 DIV with Lipofectamine 2000 (11668019, Thermo Scientific) with either FLAG-tagged *CHMP2B^Wildtype^* or *CHMP2B^Intron5^*, described previously (61) and treated with either or stavudine or lamivudine for 48 h (10 μm).

### Statistical analysis

The statistical data were performed using GraphPad Prism (6.01). Data are presented as mean values, from at least three independent biological replicates, with error bars representing the standard error of mean. All details of the tests used were outlined within each figure legend.

## Supplementary Material

figS1FortAznar_ddaa142

figS2FortAznar_ddaa142
