# Supplementary figures and images for: Retrovirus reactivation in CHMP2BIntron5 models of frontotemporal dementia
Source: Hum Mol Genet. 2020 Jul 6;29(16):2637–46. doi: 10.1093/hmg/ddaa142 (PMC7530534; doi:10.1093/hmg/ddaa142)

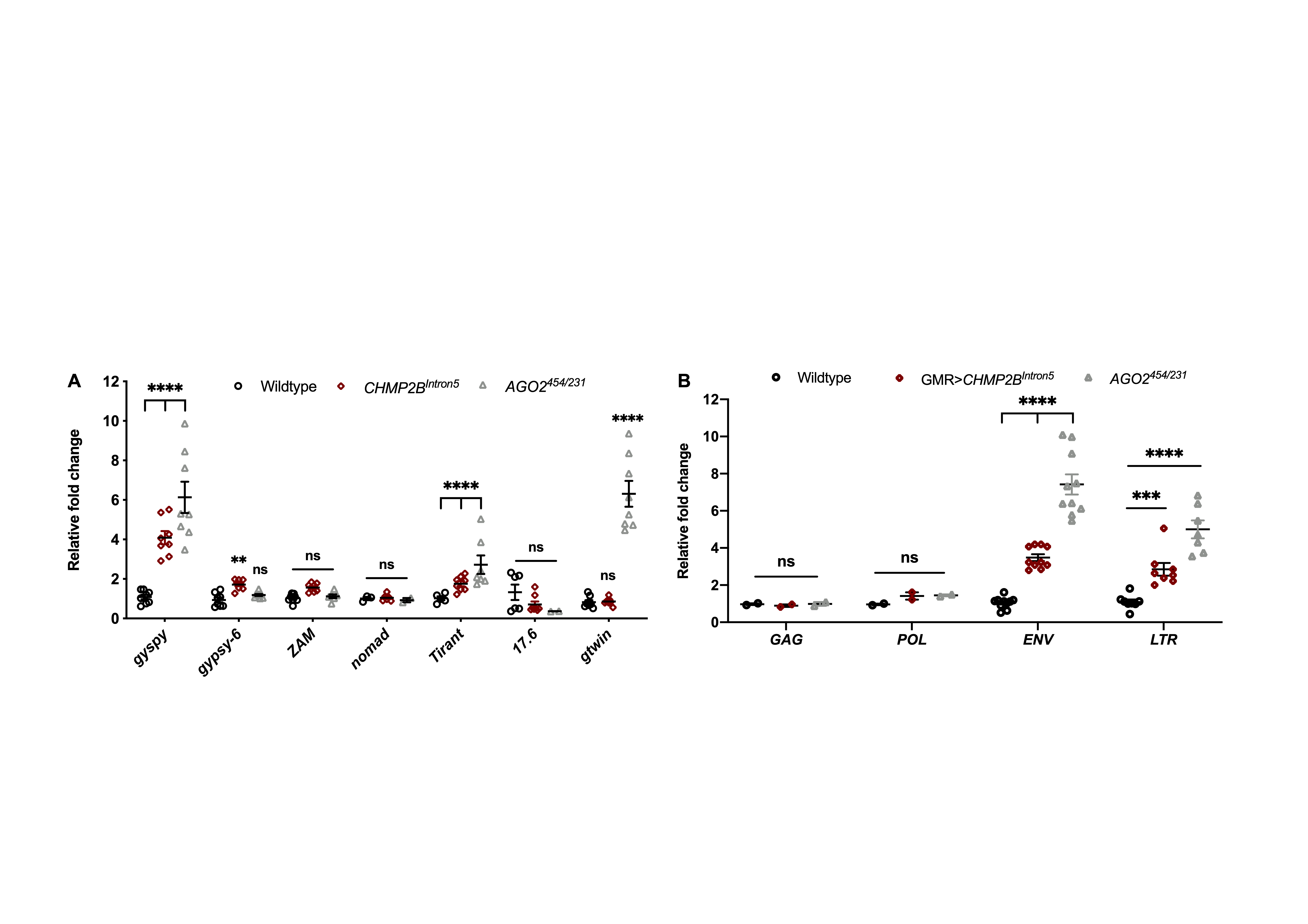

Supplement: figS1FortAznar_ddaa142 [file figs1fortaznar_ddaa142.png]

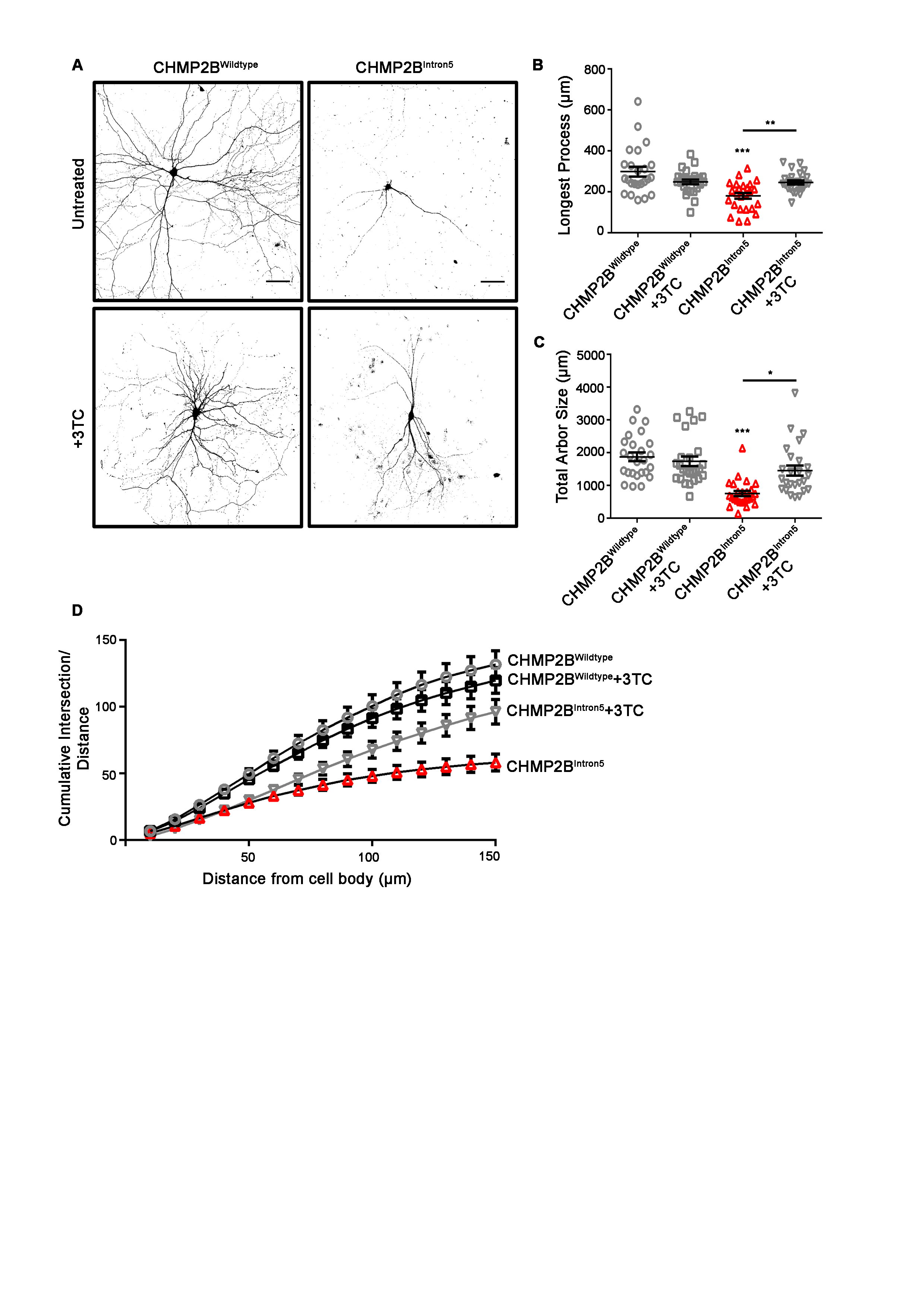

Supplement: figS2FortAznar_ddaa142 [file figs2fortaznar_ddaa142.png]
